# Persistent HyperCKemia Beyond Post-Viral Myositis: A Case of Anti-HMGCR Immune-Mediated Necrotizing Myopathy

**DOI:** 10.7759/cureus.116203

**Published:** 2026-09-14

**Authors:** Rita Xavier, Sara Silva, Rita Cunha, Angela Coelho, Carla Peixoto

**Affiliations:** 1 Internal Medicine, Centro Hospitalar do Médio Ave, Vila Nova de Famalicão, PRT; 2 Internal Medicine, Unidade Hospitalar de Famalicão, Famalicão, PRT; 3 Internal Medicine, Centro Hospitalar de Trás-os-Montes e Alto Douro, E.P.E., Chaves, PRT

**Keywords:** anchoring bias, anti-hmgcr antibody, diagnostic reasoning, dysphagia, hyperckemia, immune-mediated necrotizing myopathy, statin-associated myopathy

## Abstract

Anti-3-hydroxy-3-methylglutaryl-coenzyme A reductase (anti-HMGCR) immune-mediated necrotizing myopathy (IMNM) is a rare autoimmune myopathy that should be suspected in patients with progressive proximal muscle weakness and marked creatine kinase (CK) elevation persisting despite statin discontinuation, although distinguishing it from more common conditions such as post-viral myositis and toxic statin-associated myopathy remains a major diagnostic challenge. We report the case of a 64-year-old man receiving long-term atorvastatin therapy who presented with progressive proximal muscle weakness, dysphagia, and severe hyperCKemia following a self-limited flu-like illness, initially raising post-viral myositis as the leading hypothesis. Despite immediate statin discontinuation and aggressive intravenous hydration, severe hyperCKemia persisted during the first week of supportive management alone, and the neurological deficits did not improve, prompting further investigation and empirical immunosuppression. Muscle magnetic resonance imaging demonstrated multifocal inflammatory muscle edema, while extensive investigation for infectious, metabolic, endocrine, neoplastic, and autoimmune causes was unrevealing. Muscle biopsy showed a necrotizing myopathy with pauci-inflammatory features, and anti-HMGCR antibodies, which had not been included in the initial extended myositis panel and required a dedicated request, were subsequently identified, confirming statin-associated IMNM. Treatment with high-dose corticosteroids followed by methotrexate produced progressive biochemical improvement and complete recovery of muscle strength and swallowing function, with CK falling to 376 U/L (N 30-200 U/L) at the last follow-up (four months after admission). The principal educational value of this case lies in the diagnostic reasoning rather than in the diagnosis itself: it was the failure of the clinical and biochemical course to follow the anticipated trajectory, rather than the initial presentation, that prompted reconsideration of the working hypothesis. Dysphagia, less commonly reported in post-viral myositis and toxic statin-associated myopathy, together with persistent severe hyperCKemia despite statin withdrawal, should prompt evaluation for anti-HMGCR IMNM.

## Introduction

Statins remain the cornerstone of lipid-lowering therapy for the primary and secondary prevention of atherosclerotic cardiovascular disease. Although generally safe and well tolerated, statin-associated muscle adverse events encompass a broad clinical spectrum ranging from self-limited myalgia and toxic myopathy to, rarely, immune-mediated necrotizing myopathy (IMNM), a distinct autoimmune entity that requires early recognition and immunosuppressive treatment rather than simple withdrawal of the offending drug [1,2].

Anti-3-hydroxy-3-methylglutaryl-coenzyme A reductase (anti-HMGCR) antibody-associated IMNM is characterized by progressive symmetrical proximal muscle weakness, which may be accompanied by myalgia and dysphagia, marked serum creatine kinase (CK) elevation, and ongoing muscle fiber necrosis despite statin discontinuation [2-4]. Unlike toxic statin-associated myopathy, which generally improves after withdrawal of statin therapy, anti-HMGCR IMNM reflects a self-perpetuating autoimmune response directed against HMG-CoA reductase, resulting in persistent muscle injury [2-4]. Diagnosis relies on the integration of clinical findings with anti-HMGCR antibody testing, muscle magnetic resonance imaging (MRI), and muscle biopsy, with the latter typically demonstrating prominent myofiber necrosis with minimal inflammatory infiltrates [1,3,5,6].

Because its initial presentation frequently overlaps with more common conditions, including toxic statin-associated myopathy and post-viral myositis, the diagnosis may be delayed until the clinical evolution becomes incompatible with these alternatives [2,4,7]. Persistent muscle weakness and sustained severe hyperCKemia despite statin withdrawal should therefore prompt clinicians to reconsider the initial diagnostic hypothesis and investigate for anti-HMGCR IMNM [1,2,4].

Statin-associated anti-HMGCR IMNM is itself well characterized, and the contribution of the present report does not lie in the diagnosis. We present it because of the diagnostic reasoning that led to it: a plausible initial hypothesis, a clinical course that failed to conform to it, and the deliberate reconsideration that followed. The case also illustrates a practical pitfall that is easily overlooked, namely that a negative extended myositis antibody panel does not exclude anti-HMGCR IMNM when the antibody is not among the specificities tested.

## Case presentation

A 64-year-old man presented to the Emergency Department with a two-week history of progressive proximal muscle weakness and dysphagia. His medical history included type 2 diabetes mellitus, dyslipidemia treated with atorvastatin (20 mg/day since 2022), previous ischemic cerebrovascular disease, and parkinsonism under diagnostic investigation. He had been fully independent before symptom onset (Clinical Frailty Scale 3) [8,9] and was working as a train operator.

Three weeks before admission, he developed fever and a non-productive cough that resolved after symptomatic treatment. Approximately one week later, he experienced progressive weakness initially affecting the left hemibody, which was attributed to his previous cerebrovascular disease, followed by symmetrical proximal weakness involving all four limbs. Dysphagia for solid foods developed shortly thereafter. He denied myalgia, recent strenuous exercise, trauma, alcohol consumption, illicit drug use, herbal supplements, or recent medication changes.

On admission, neurological examination demonstrated severe symmetrical proximal tetraparesis (Medical Research Council grade 3/5), while distal muscle strength, cognition, and sensory examination were preserved - a pattern inconsistent with a central neurological cause. No cutaneous manifestations suggestive of inflammatory myopathy were observed.

Initial laboratory evaluation revealed severe rhabdomyolysis, with a CK concentration of 22,860 U/L (reference range: 30-200 U/L), myoglobin 11,876 ng/mL (reference range: 17.4-105.7 ng/mL), aspartate aminotransferase 649 U/L (reference range: 0-45 U/L), alanine aminotransferase 473 U/L (N 0-55 U/L), and lactate dehydrogenase 1,156 U/L (reference range: 125-220 U/L), while renal function remained preserved despite the marked muscle injury (Table 1).

**Table 1:** Baseline clinical characteristics and laboratory findings at presentation ALT, alanine aminotransferase; AST, aspartate aminotransferase; CK, creatine kinase; MRC, Medical Research Council

Variable	Finding
Age/sex	64 years / male
Relevant medical history	Type 2 diabetes mellitus; dyslipidemia; previous ischemic stroke; parkinsonism under diagnostic investigation
Chronic medication	Atorvastatin (since 2022), aspirin, metformin, levodopa/carbidopa, rotigotine
Presenting symptoms	Progressive proximal muscle weakness, dysphagia for solids
Preceding illness	Flu-like syndrome (fever and dry cough) 3 weeks before admission
Physical examination	Symmetric proximal tetraparesis (MRC 3/5), no skin manifestations
Peak CK	20,940 U/L
Peak myoglobin	12,697 ng/mL
Peak AST/ALT	649 / 473 U/L
Renal function	Preserved
Initial differential diagnosis	Post-viral myositis; statin-associated toxic myopathy

Given the recent viral syndrome, post-viral myositis was initially considered the most likely diagnosis. Atorvastatin was discontinued immediately on admission, and aggressive intravenous hydration (approximately 3 L/day) was maintained. Nevertheless, CK and myoglobin values showed no meaningful decline during the first week of supportive therapy alone, and both proximal weakness and dysphagia persisted (Table 2). This clinical evolution was considered atypical for either post-viral myositis or self-limited toxic statin myopathy, prompting further diagnostic investigation.

**Table 2:** Serial laboratory parameters, corticosteroid dose, and major clinical milestones during hospitalization and follow-up Day 0 corresponds to the day of emergency department admission. The corticosteroid column reports the daily dose of oral prednisolone in milligrams; no intravenous methylprednisolone was administered. "None" indicates that corticosteroid therapy had not yet been started, whereas a dash (-) indicates that the parameter was not measured or not available for that day. Reference ranges: CK 30-200 U/L; myoglobin 17.4-105.7 ng/mL; AST 0-45 U/L; ALT 0-55 U/L. ALT, alanine aminotransferase; anti-HMGCR, anti-3-hydroxy-3-methylglutaryl-coenzyme A reductase; AST, aspartate aminotransferase; CK, creatine kinase

Day	CK (U/L)	Myoglobin (ng/mL)	AST (U/L)	ALT (U/L)	Prednisolone (mg/day)	Major clinical event
D0	22,860	11,876	649	473	None	Emergency department admission; atorvastatin withdrawn; intravenous hydration started
D1	20,940	10,541	578	420	None	Admission to the internal medicine ward
D2	17,848	12,676	491	109	None	-
D3	15,275	8,202	427	64	None	-
D7	14,511	12,697	399	92	70	Oral prednisolone 1 mg/kg/day started
D8	10,376	8,306	302	146	70	-
D10	7,978	10,276	262	289	70	-
D11	6,726	7,285	-	-	60	-
D14	5,277	7,161	182	104	50	-
D16	4,045	7,549	151	107	50	-
D20	-	-	-	-	40	-
D22	4,789	7,229	-	-	40	Anti-HMGCR antibody testing requested; sample collected
D23	2,697	3,976	-	-	40	-
D25	2,242	3,150	-	-	40	-
D30	1,419	2,566	65	224	30	-
D31	-	-	-	-	30	Muscle biopsy (right rectus femoris)
D33	1,714	2,541	82	86	20	Discharge
D35	-	-	-	-	-	Anti-HMGCR antibody reported positive (107 AU; threshold 20 AU)
D73	-	-	41	38	15	Methotrexate 7.5 mg/week started
D102	-	-	-	-	10	Methotrexate increased to 10 mg/week
D116	376	153	21	36	10	Last follow-up

An extensive evaluation excluded infectious, endocrine, metabolic, autoimmune, and paraneoplastic causes. Thyroid function, vitamin B12 and folate concentrations, serum protein electrophoresis, tumor markers, viral serologies, and a comprehensive myositis autoantibody panel were unrevealing. Computed tomography of the neck, chest, abdomen, and pelvis showed no evidence of occult malignancy.

Magnetic resonance imaging of the lower limbs demonstrated multifocal muscle edema involving several muscle groups, with the most pronounced inflammatory changes affecting the right rectus femoris, which was therefore selected for muscle biopsy (Figure 1).

**Figure 1:**
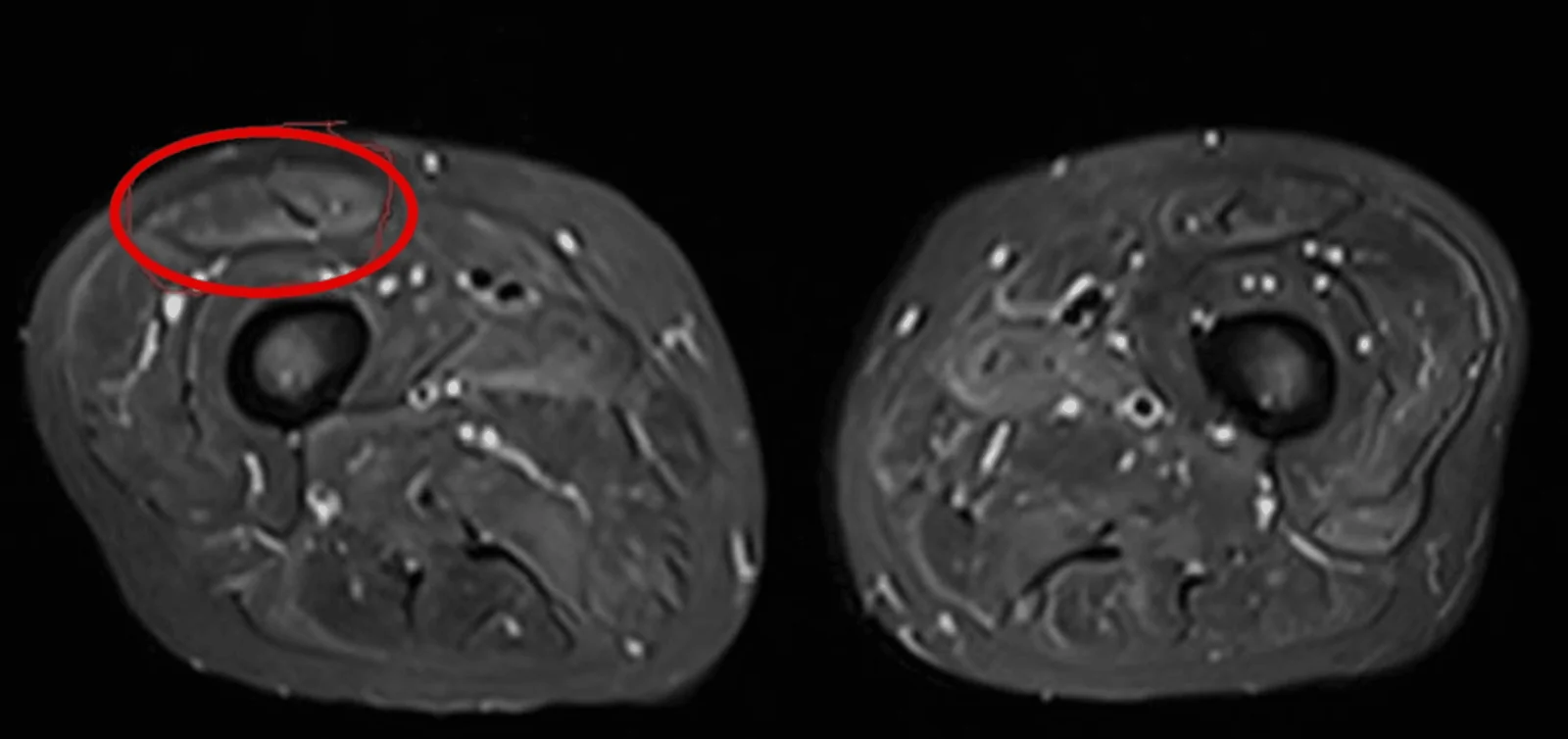
Muscle MRI in anti-HMGCR immune-mediated necrotizing myopathy Axial STIR thigh MRI shows hyperintense muscle edema in the right antero-medial compartment (red circle), indicating active myositis. anti-HMGCR, anti-3-hydroxy-3-methylglutaryl-coenzyme A reductase

Because of the persistent severe hyperCKemia, the lack of clinical improvement, and MRI findings suggestive of inflammatory myopathy, empirical treatment with oral prednisolone 1 mg/kg/day (70 mg daily) was initiated on hospital day 7 and subsequently tapered according to the clinical and biochemical response (Table 2). No intravenous methylprednisolone pulses were administered at any point.

In view of the refractory course, dedicated testing for anti-HMGCR antibodies was requested on hospital day 22, as this specificity had not been covered by the initial extended panel. Serological testing and muscle biopsy were therefore pursued in parallel rather than sequentially. Muscle biopsy had not been performed at presentation because post-viral myositis was considered the most likely diagnosis and a period of observation was judged appropriate; once the course proved refractory, the procedure was further deferred by the scheduling of muscle MRI, which was undertaken first in order to guide the biopsy site. The biopsy was therefore performed on hospital day 31.

Histopathological examination of the right rectus femoris demonstrated an active necrotizing myopathy with pauci-inflammatory features, characterized by scattered necrotic and regenerating muscle fibers, diffuse sarcolemmal overexpression of major histocompatibility complex class I (MHC-I), focal membrane attack complex (C5b-9) deposition, and sparse endomysial inflammatory infiltrates composed predominantly of macrophages and T lymphocytes, with no significant perifascicular atrophy or inclusion bodies. These findings were highly suggestive of IMNM (Figures 2, 3).

**Figure 2:**
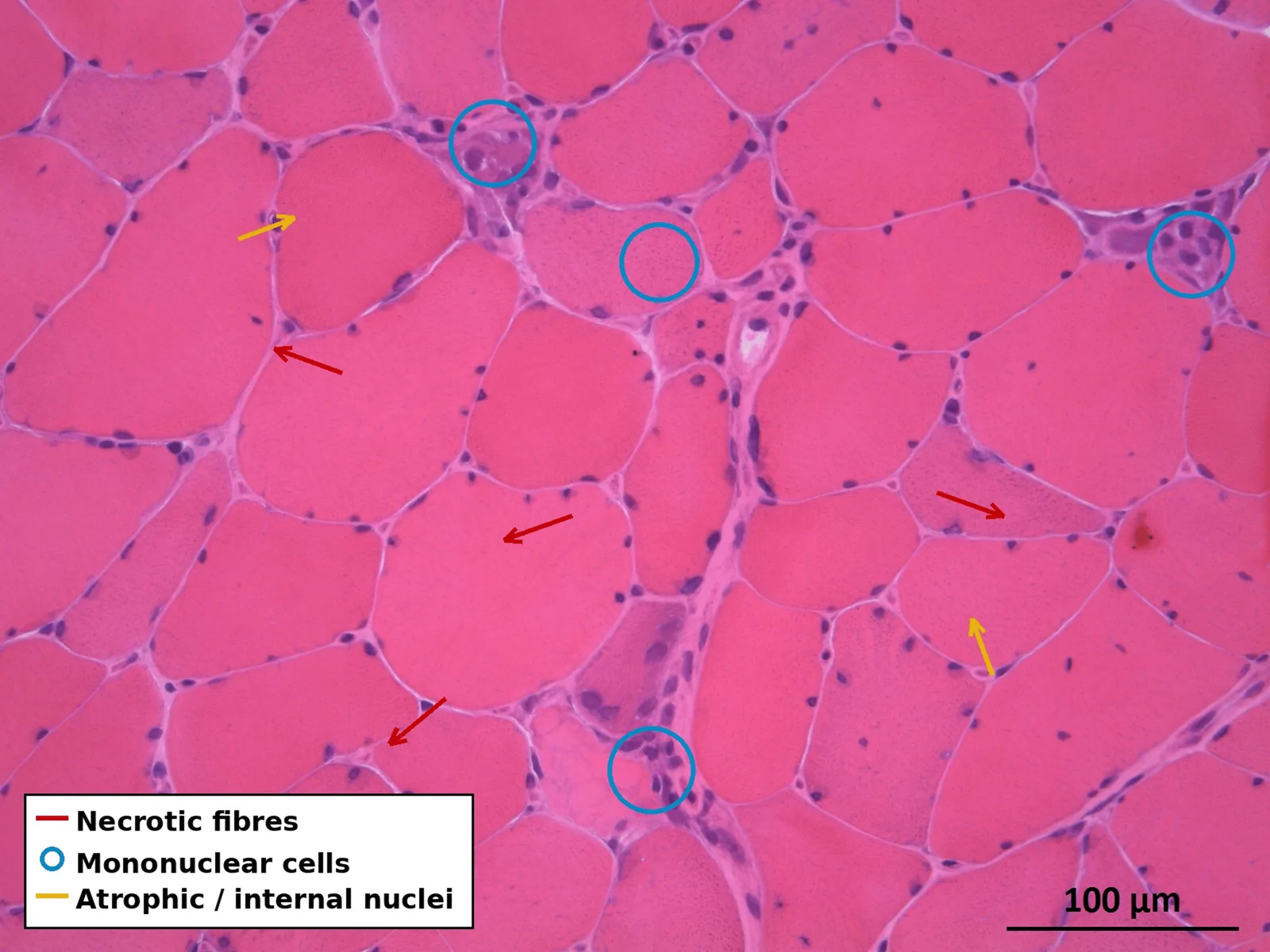
Muscle biopsy findings in anti-HMGCR immune-mediated necrotizing myopathy H&E (bar 100 µm): scattered necrotic fibres (red arrows), sparse mononuclear infiltrate (circles), internalized nuclei (yellow arrows) - pauci-inflammatory necrotizing pattern. anti-HMGCR, anti-3-hydroxy-3-methylglutaryl-coenzyme A reductase

**Figure 3:**
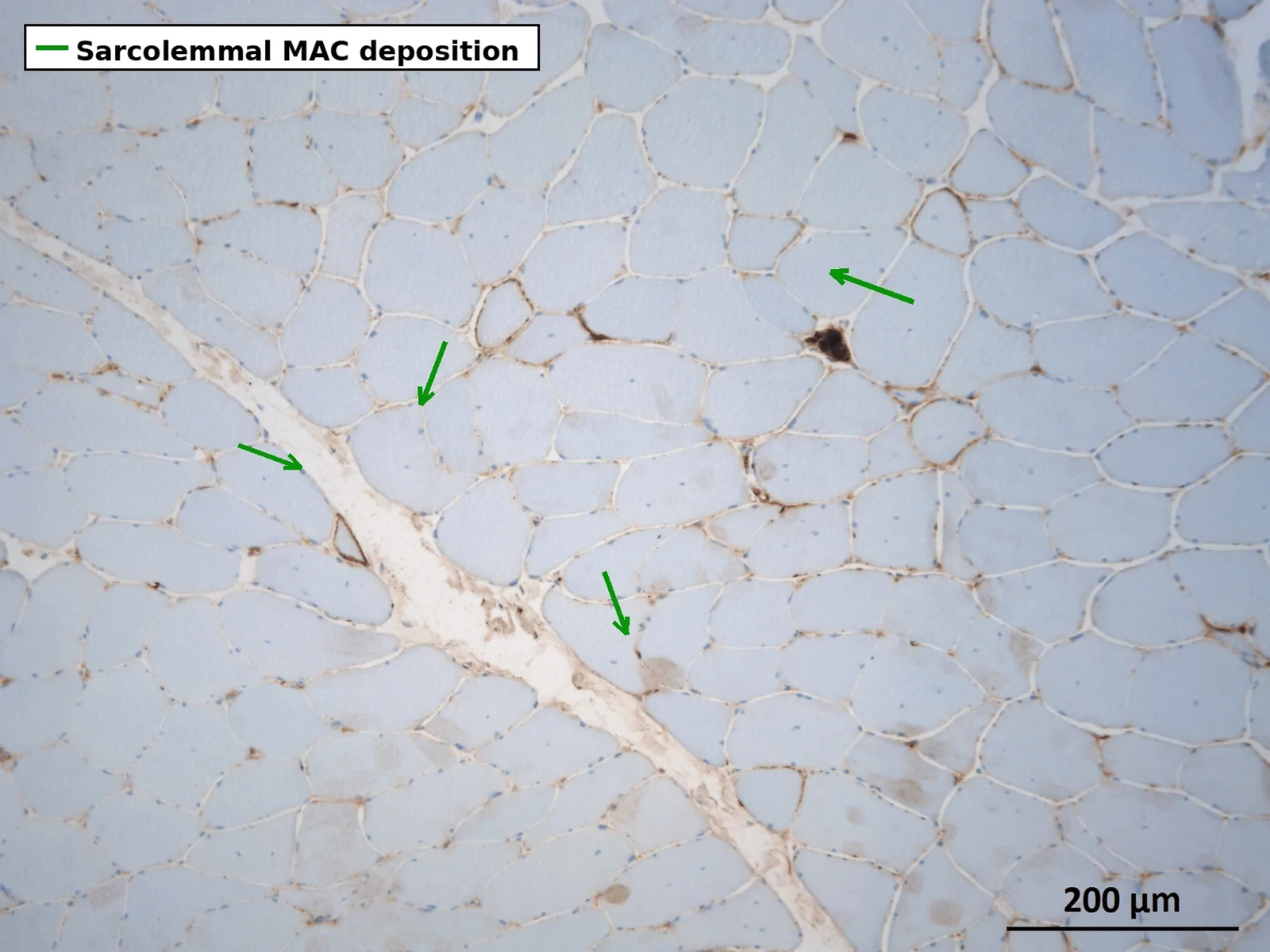
Muscle biopsy findings in anti-HMGCR immune-mediated necrotizing myopathy MAC/C5b-9 immunohistochemistry (bar 200 µm): sarcolemmal MAC deposition on non-necrotic fibers (green arrows). anti-HMGCR, anti-3-hydroxy-3-methylglutaryl-coenzyme A reductase; MAC, membrane attack complex

Anti-HMGCR antibodies were reported as positive by enzyme immunoassay, with a quantitative result of 107 arbitrary units (AU) against a laboratory positivity threshold of 20 AU, that is, more than five times the cut-off value. The result became available on hospital day 35, four days after the muscle biopsy and two days after discharge, and established the diagnosis of statin-associated anti-HMGCR IMNM.

The patient underwent intensive physical rehabilitation and speech therapy throughout hospitalization. Progressive biochemical improvement followed the initiation of immunosuppressive therapy, accompanied by gradual recovery of muscle strength and swallowing function. At discharge on day 33, muscle strength had improved to Medical Research Council grade 4/5, dysphagia had markedly improved, and CK had decreased to 1,714 U/L.

Methotrexate (7.5 mg/week) was introduced by the neurology team at the first outpatient visit after serological confirmation (day 73), at which point transaminases had normalized (AST 41 U/L, ALT 38 U/L) and hepatic safety monitoring had become interpretable. The starting dose reflected conventional incremental titration and was increased to 10 mg/week on day 102, while prednisolone was progressively tapered. At the last evaluation, on day 116 and approximately four months after admission, the patient had achieved complete functional recovery, with normal muscle strength, independent ambulation, and complete resolution of dysphagia; CK had decreased to 376 U/L, remaining marginally above the upper limit of normal. The full clinical course is summarized in Figure 4.

**Figure 4:**
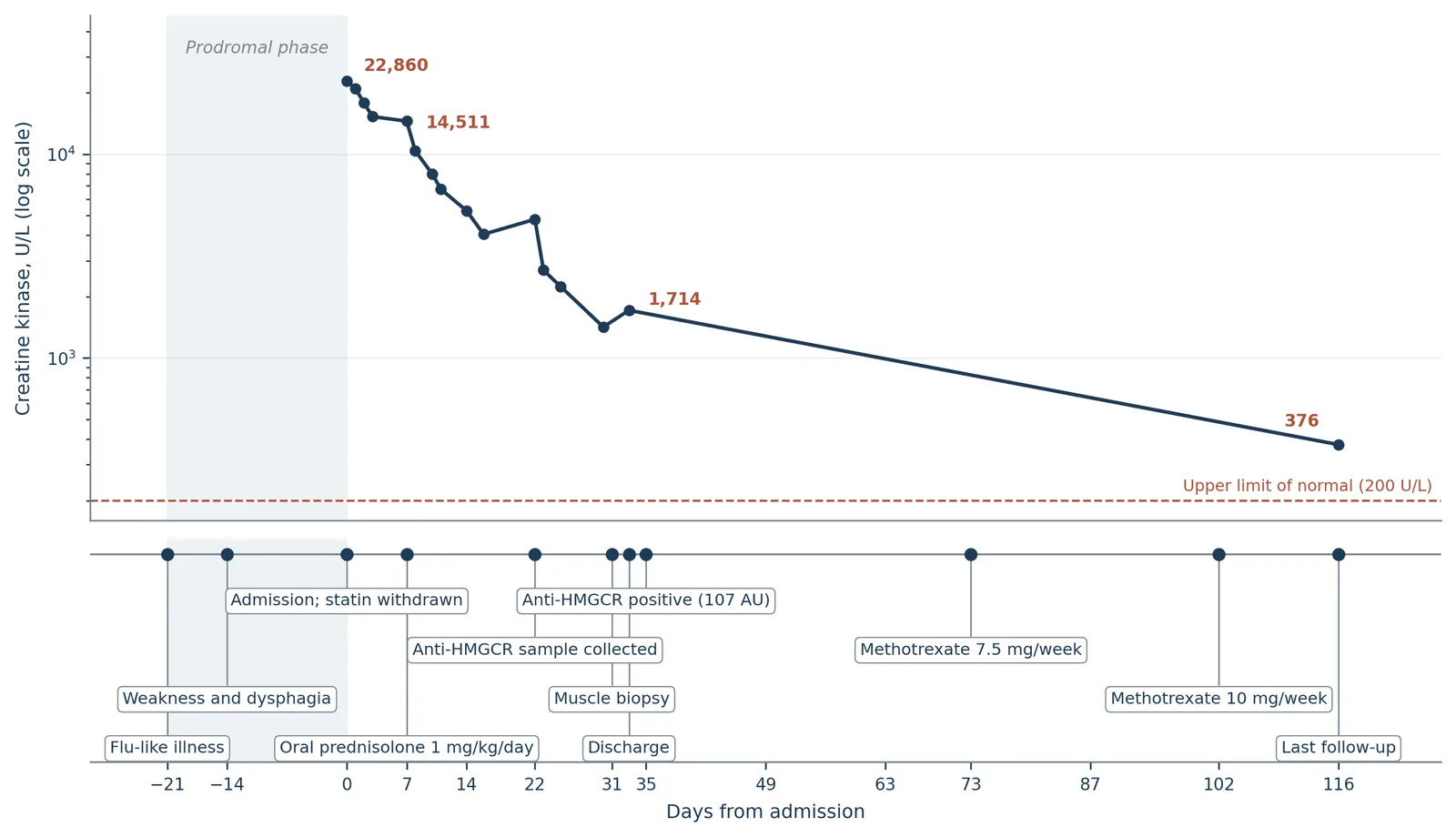
Timeline of the clinical course and serum creatine kinase trajectory Upper panel: serial serum creatine kinase concentrations plotted on a logarithmic scale against days from admission; the dashed line marks the upper limit of the laboratory reference range (200 U/L). Selected values are annotated. Lower panel: corresponding clinical, diagnostic, and therapeutic milestones. Day 0 corresponds to the day of emergency department admission, and negative values denote the period preceding admission; the shaded area indicates the prodromal phase. Creatine kinase remained severely elevated throughout the first week despite atorvastatin withdrawal and intravenous hydration and began to fall progressively only after the introduction of oral prednisolone on day 7. Anti-HMGCR antibody testing was requested on day 22, nine days before the muscle biopsy, and the positive result became available on day 35. AU, arbitrary units; anti-HMGCR, anti-3-hydroxy-3-methylglutaryl-coenzyme A reductase The figure was created using Claude Opus 5 (Anthropic)

## Discussion

IMNM is a rare subtype of idiopathic inflammatory myopathy characterized by progressive symmetrical proximal muscle weakness, marked serum CK elevation, myofiber necrosis with minimal inflammatory infiltrates on muscle biopsy, and a strong association with myositis-specific autoantibodies, particularly anti-HMGCR and anti-signal recognition particle (SRP) antibodies [1,4-6]. Among patients with anti-HMGCR IMNM, long-term statin exposure is common, although the disease may also occur in statin-naive individuals [2-4]. Unlike toxic statin-associated myopathy, IMNM results from a self-sustaining autoimmune response against HMG-CoA reductase, which explains the persistence of muscle injury despite withdrawal of the offending drug [2-4].

The principal educational value of this case lies not in the diagnosis itself but in the diagnostic reasoning that ultimately led to it. At presentation, post-viral myositis appeared to be the most plausible diagnosis given the recent flu-like illness, the absence of cutaneous manifestations suggestive of inflammatory myopathy, and the lack of previous statin intolerance despite several years of atorvastatin therapy. In routine clinical practice, this would reasonably represent the leading initial hypothesis. The subsequent course, however, progressively challenged that assumption. Despite immediate statin discontinuation and aggressive intravenous hydration, CK fell only from 22,860 U/L to 14,511 U/L over the first week, and severe hyperCKemia therefore persisted, while severe proximal weakness and dysphagia remained unchanged. This evolution was inconsistent with the expected natural history of both post-viral myositis and toxic statin-associated myopathy, in which biochemical improvement is typically accompanied by parallel clinical recovery after supportive treatment and withdrawal of the offending agent [2,4,7]. Rather than the initial presentation itself, it was the failure of the disease to follow the anticipated trajectory that prompted reconsideration of the diagnosis and further investigation.

Dysphagia deserves particular emphasis, as it is a potentially discriminating feature. It has been reported in roughly one-quarter to one-third of patients with anti-HMGCR IMNM, affecting 28.8% of the 691 patients included in a pooled analysis of published cohorts [10], whereas bulbar involvement is less commonly described in toxic statin-associated myopathy and in post-viral myositis [2,7]. This is a difference of relative frequency rather than an absolute distinction, and dysphagia alone cannot establish or exclude the diagnosis. Although dysphagia and reduced mobility may also occur in parkinsonian syndromes, in this patient, both symptoms were of recent onset, developed concurrently with proximal tetraparesis, and resolved completely with immunosuppression, supporting a myopathic rather than an extrapyramidal origin. In retrospect, dysphagia at presentation was an early clue favoring an immune-mediated process, and its recognition may prompt earlier consideration of IMNM.

The preceding viral syndrome may have represented either a coincidental event or a potential trigger for autoimmune activation. Viral infections have been proposed as environmental triggers for autoimmune diseases in genetically susceptible individuals, although evidence supporting a direct causal role in anti-HMGCR IMNM remains limited [1,11]. Regardless of its pathogenic significance, the temporal association probably contributed to diagnostic anchoring and delayed consideration of an IMNM.

Beyond post-viral myositis and toxic statin-associated myopathy, the differential diagnosis of severe hyperCKemia with proximal weakness includes other idiopathic inflammatory myopathies, namely dermatomyositis, antisynthetase syndrome, and anti-SRP IMNM, which were considered unlikely given the absence of cutaneous, articular, and pulmonary manifestations and the results of the extended myositis-specific antibody panel. Endocrine and metabolic causes such as hypothyroidism and severe electrolyte disturbance, infectious myositis, and paraneoplastic myopathy were excluded by the corresponding investigations. Inherited disorders, including limb-girdle muscular dystrophy and metabolic myopathies such as McArdle disease, were considered improbable in view of the patient's age, previously normal muscle function and occupational activity, and the acute onset after years of uneventful statin exposure; genetic testing was therefore not pursued.

The diagnosis of anti-HMGCR IMNM relies on the integration of clinical findings with serological testing, muscle MRI, and histopathological examination [1,4,6]. The extended myositis antibody panel performed during the initial work-up was negative; anti-HMGCR antibodies, however, were not included in that panel and required a separate, dedicated request, which was made on hospital day 22, nine days before the muscle biopsy, once the course had proved refractory. This point deserves emphasis because a negative myositis panel may be misinterpreted as excluding an autoimmune myopathy when the relevant antibody has simply not been tested [3,12]. Clinicians should therefore verify which specificities a given panel covers before drawing conclusions from a negative result, particularly when the clinical picture remains suggestive. In this patient, the antibody was strongly positive, at 107 AU against a threshold of 20 AU; anti-HMGCR levels have been shown to correlate with CK concentrations and with muscle strength at presentation and to decline with immunosuppressive treatment, although they rarely normalize [13].

Muscle MRI demonstrated multifocal inflammatory muscle edema and guided the selection of the right rectus femoris for biopsy, maximizing diagnostic yield [6]. Histopathological examination revealed a necrotizing myopathy with pauci-inflammatory features, diffuse sarcolemmal MHC-I overexpression, and scattered necrotic fibers, findings characteristic of IMNM [1,5,6]. The biopsy was performed on day 31, that is, 24 days after corticosteroid therapy had been started, a delay that reflected both the initial diagnostic confidence in post-viral myositis and the subsequent scheduling of muscle MRI. Prior corticosteroid exposure may attenuate inflammatory infiltrates and reduce diagnostic yield, and the sparse endomysial infiltrate observed here should be interpreted with that caveat. Nevertheless, the defining features of IMNM, namely myofiber necrosis and regeneration, diffuse sarcolemmal MHC-I overexpression, and C5b-9 deposition, remained clearly demonstrable, and the biopsy was diagnostic. That these findings persisted after nearly a month of immunosuppression arguably reinforces rather than weakens the diagnosis. Electromyography was not performed because of local unavailability; the combination of the clinical presentation, imaging findings, positive anti-HMGCR antibodies, and muscle biopsy nonetheless provided sufficient diagnostic confidence and would not have altered therapeutic management.

Early immunosuppressive therapy is essential in anti-HMGCR IMNM because statin discontinuation alone is insufficient to halt disease progression [2,4,14-17]. Current expert recommendations advocate high-dose corticosteroids combined with the early introduction of a steroid-sparing agent such as methotrexate or azathioprine, while intravenous immunoglobulin (IVIG) is increasingly used in this subtype, either as an adjunct in severe or refractory disease or as first-line monotherapy [1,4,14,15,18,19]. In our patient, corticosteroids were started empirically on day 7, before histological and serological confirmation, and were followed by a prompt and sustained biochemical and functional response. IVIG was therefore not administered: the patient improved both clinically and biochemically on corticosteroids alone and at no point required escalation of therapy. We note that no universally accepted criteria define refractory disease in this setting and that the decision was accordingly a pragmatic one, based on the observed response rather than on any formal threshold. Methotrexate was introduced by the neurology team at the first outpatient visit after serological confirmation, at a starting dose of 7.5 mg weekly with subsequent incremental escalation, once transaminases had normalized and hepatic safety monitoring had become interpretable. Whether earlier IVIG or earlier introduction of a steroid-sparing agent would have shortened the time to biochemical remission cannot be determined from a single case and represents a limitation of this report.

At the last evaluation, four months after admission, the patient had achieved complete functional recovery, although CK had not fully normalized (376 U/L). Persistent mild hyperCKemia is common in anti-HMGCR IMNM, and clinically significant relapses are recognized during tapering of immunosuppression; whether a persistently elevated CK predicts relapse remains unresolved, but it supports continued clinical and biochemical surveillance [20]. The relatively short duration of follow-up is a further limitation of this report. The favorable outcome nonetheless reinforces the importance of early recognition and prompt initiation of immunosuppressive therapy before irreversible muscle damage occurs.

## Conclusions

Anti-HMGCR IMNM should be considered in patients presenting with progressive proximal muscle weakness and persistent marked hyperCKemia despite statin discontinuation, particularly when the clinical course is inconsistent with post-viral myositis or toxic statin-associated myopathy. This case illustrates the importance of reassessing the initial diagnostic hypothesis when the expected biochemical and functional recovery does not occur, as well as the risk of anchoring on a plausible but incorrect explanation. Persistent hyperCKemia should be regarded not only as a marker of ongoing muscle injury but also as a diagnostic clue prompting further investigation for an immune-mediated myopathy. A negative extended myositis antibody panel does not exclude the diagnosis when anti-HMGCR is not among the specificities tested, and dedicated testing should be requested whenever clinical suspicion persists. Early serological confirmation and muscle biopsy, followed by timely immunosuppressive therapy, are essential to optimize functional recovery and prevent irreversible muscle damage. In this patient, complete clinical and functional recovery was achieved, while biochemical improvement remained ongoing, with CK still marginally above the reference range at the last follow-up, a distinction that supports continued surveillance.
